# Endovascular retrieval of a migrated contraceptive implant into the pulmonary artery : case report and review of literature

**DOI:** 10.1186/s42155-024-00450-w

**Published:** 2024-04-06

**Authors:** Rémi Grange, Nicolas Magand, Nathalie Grand, Stéphanie Leroy, Thomas Corsini, Kasra Azarnoush, Sylvain Grange

**Affiliations:** 1 Department of Radiology, University Hospital of Saint-Etienne, Avenue Albert Raimond, 42270 Saint-Priest-en-Jarez, France; 2 Department of Anesthesiology, University Hospital of Saint-Etienne, Saint-Priest-en-Jarez, France; 3 Department of Gynecology, University Hospital of Saint-Etienne, Saint-Priest-en-Jarez, France; 4 Department of Cardiac Surgery, University Hospital of Saint-Etienne, Saint-Priest-en-Jarez, France

**Keywords:** Pulmonary artery, Foreign body, Endovascular approach

## Abstract

**Background:**

The migration of contraceptive devices into pulmonary arteries is extremely rare, reported to be 1 in 100,000.

**Case presentation:**

A 19-year-old female presented no sensation of a contraceptive implant in her arm which had been placed one year prior. A CT scan confirmed that the implant had migrated into the left lower segmentary pulmonary artery. After a multidisciplinary meeting, an endovascular approach was attempted. Following right femoral venous access, a 8F NeuronMax® introducer was placed into the left pulmonary artery under fluoroscopic guidance. The contraceptive device was removed using a 25-mm loop snare, with a proximal capture technique. The patient was discharged the following day, with no reported complications.

**Conclusion:**

In cases of contraceptive device migration, the first medical decision involves deciding between removal or 'watching and waiting'. Previous reports describe two removal options: endovascular or surgical approaches. Fourteen reports have been published, with high technical success and low rates of complications. The loop snare technique is described as the optimal technique for an endovascular approach. Due to their invasive nature, surgical approaches should be reserved for cases of endovascular removal failure, after evaluating risks and benefits.

**Graphical Abstract:**

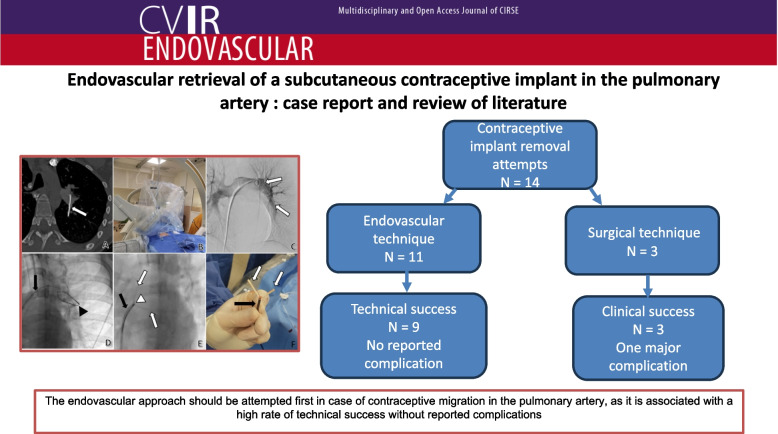

## Background

The Nexplanon® device is a subcutaneous contraceptive etonostrogel rod, measuring 4 cm × 3 mm, which is implanted in the arm above the medial epicondyle. It is mainly used for contraception or to treat menorrhagia. Complications reported with this type of contraception include dysmenorrhea (2.8%), dyspareunia (1.6%), cervicitis (2.0%), or the occurrence of a normal pregnancy (1.5%) [1]. One of the complications involves the migration of the device. The device usually migrates less than 2 cm within three months post-insertion. Migration into the axillary vein [2] or pulmonary artery is very rare. The incidence of migration into the pulmonary artery is reported to be 1 in 100,000 [3]. There is currently no specific recommendation regarding the management of migration within the pulmonary artery. The device can migrate into an axillary vein or the pulmonary artery, segmentally or sub-segmentally. This case report describes the endovascular removal of a migrated subcutaneous contraceptive implant into the left pulmonary artery using a loop snar and review all reported cases from the literature.

## Case presentation

**Fig. 1:**
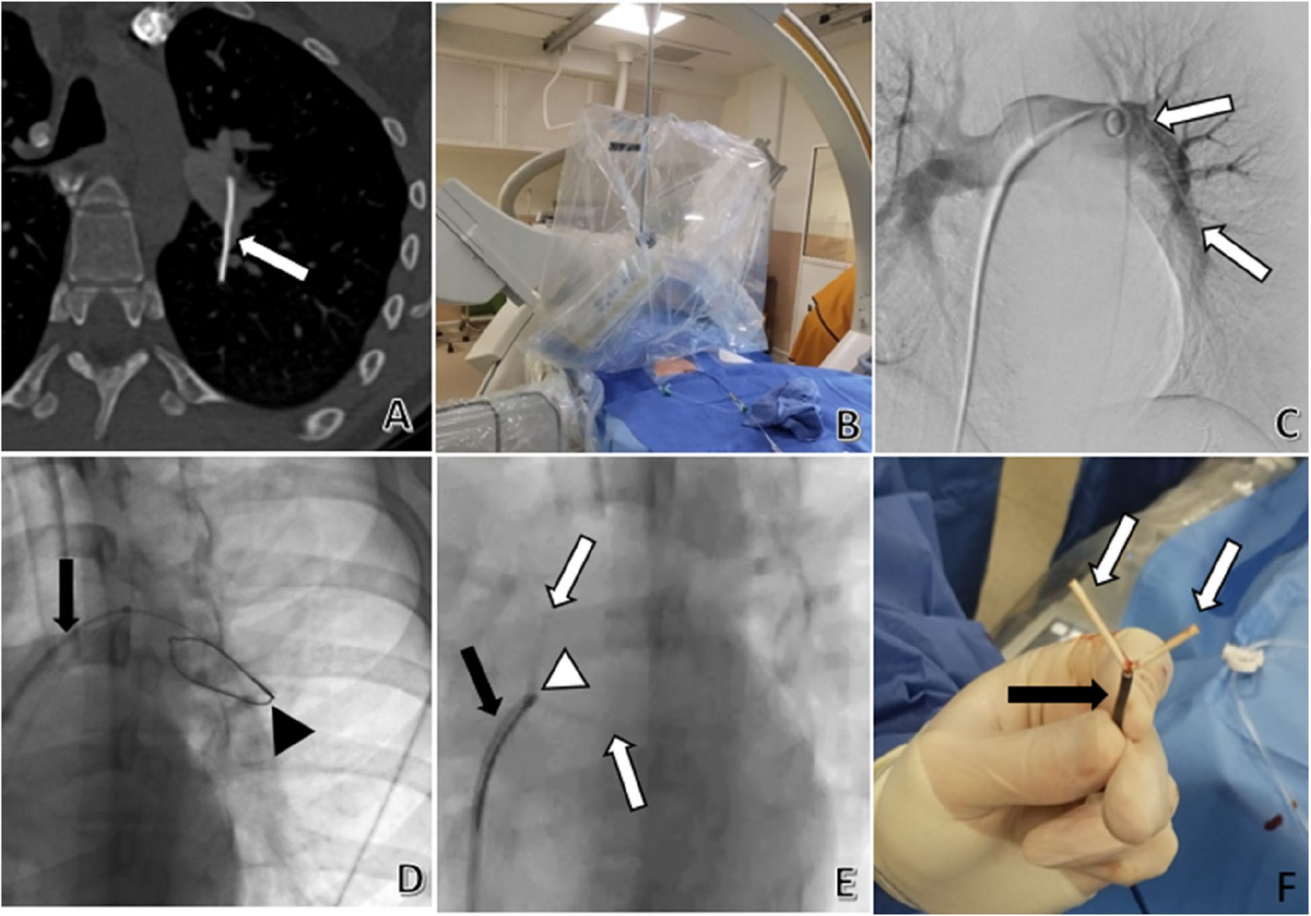
Endovascular retrieval of a contraceptive implant in the pulmonary artery. **A** A CT scan in the axial section showing a contraceptive device partially located in a left lower lobe segmental artery and the left pulmonary artery (white arrow). **B** The patient was placed in a dorsal decubitus position in a biplane interventional room, using both left and right anterior obliquities. **C** After a right femoral approach, an angiogram using a 5F pigtail showed the contraceptive device (white arrows), with no visible thrombosis. **D** A 8F introducer was placed into the left pulmonary artery (black arrow), allowing insertion of a 6F sheath and a loop snare (black arrowhead). **E** After pulling the contraceptive device (white arrows) with the loop snare (white arrowhead), it was then locked in front of the sheath and the introducer (black arrow). **F** After removing the entire introducer, the contraceptive device (white arrows) was trapped by the loop snare (black arrow)

A 19-year-old female had a subcutaneous implant placed one year prior to a visit to her physician. During the implantation of the device, abnormal cutaneous bleeding was noted. After the physician was unable to locate the implant, an ultrasound was performed. The ultrasound was unable to locate the contraceptive device. A thoracic CT scan revealed that the implant had migrated into the lower left lobular segmental pulmonary artery. The patient reported no symptoms related to the migration of the implant. After a multidisciplinary consultation involving a cardiac surgeon, anaesthesiologists, a gynaecologist, a cardiologist, and an interventional radiologist, an endovascular approach was considered. The patient underwent a preoperative consultation with a gynaecologist, an anaesthesiologist, and an interventional radiologist to explain the removal procedure and the known risks of pulmonary arterial catheterization. The procedure planning included the de-sterilization of a Nexplanon® to assess its flexibility. The team decided to perform the removal under bi-planar fluoroscopic guidance, as routinely done in our department for foreign body retrieval. Both right and left anterior obliquities were chosen to ideally expose the contraceptive implant (Fig. 1). The procedure was conducted under general anaesthesia. Following a right femoral venous puncture under ultrasound guidance, a long 8F NeuronMax® introducer (Penumbra, Inc., Alameda, CA, USA) was placed under fluoroscopic guidance into the right inferior vena cava. The left pulmonary artery was catheterized using a 5F 145° angled Pigtail catheter (Merit Medical, UT, USA). An angiogram confirmed the position of the contraceptive implant, without thrombosis. After guide exchange with a stiff guide wire (Terumo, Tokyo, Japan), the NeuronMax® catheter was subsequently advanced to the left pulmonary artery, just upstream of the foreign body. A 25-mm diameter loop snare (One Snare®, Merit Medical, UT, USA) was deployed. Once captured, the contraceptive implant was removed under fluoroscopic guidance without removing it into the NeuronMax® catheter. The procedure lasted 60 min. The fluoroscopic dose was 261 mGy, and fluoroscopy time was 10 min. The following day, a thoracic CT scan showed no procedure-related complications, and the patient was discharged. The patient did not receive any medical treatment before, during, or after the procedure.

## Discussion

The reasons for contraceptive device migration into the pulmonary artery are not fully understood, although intravascular migration during implantation is the most commonly proposed hypothesis. Migration is mostly asymptomatic but can lead to dyspnoea, pleural effusion [4], or pneumothorax [2].

The first medical decision involves deciding between removal [5, 6] or 'watching-and-waiting' [7, 8]. The decision for removal should be discussed with the patient due to the asymptomatic nature of device migration. In the literature, contraceptive implant migration has not been associated with thrombotic complications. The main arguments for the ‘watch-and-wait’ approach include peripheric location with patient’s refusal of surgery [8], a risk–benefit balance deemed unfavourable for both approaches [7], or the failure of the endovascular attempt and patient’s refusal of surgery [9]. Potential complications of pulmonary catheterization include possible perforation of the vessel wall, injury to the tricuspid valve, arrhythmias, artery spasm, thrombosis of the pulmonary artery, and hematoma at the puncture site [8]. The potential complication of removal is injury to a pulmonary arterial branch, with alveolar haemorrhage. We recommend direct catheterization of the pulmonary artery with an angled Pigtail catheter, without using a guide wire. Indeed, the guide wire may pass between the cordage and the heart wall, potentially causing rupture of cordage when advancing the catheter over the guide wire. Given the potential risk of such a procedure, we recommend performing it under general anesthesia. The presence of an anesthesiologist specialized in cardiac surgery, and a cardiothoracic surgeon on-site is desirable. Furthermore, given the asymptomatic nature of most reported cases, we recommend avoiding any insistence on removing the device in case of technical failure. In the present case, the patient’s young age and the partial location in the left pulmonary artery prompted us to attempt retrieval.

Previous reporting describes the two main options for contraceptive device removal: endovascular approaches [5, 9–17] and surgical approaches [4, 6, 18, 19]. In the literature, we found 14 reports of removal attempts as the first therapeutic option, 4 of which were done surgically and 10 endovascularly (Table 1). Two endovascular techniques have been described for subcutaneous device removal: the aspiration technique [14] and the loop snare technique [5, 9–13, 15–17]. The loop snare technique is the first choice to attempt removal of an intravascular foreign body. Loop snares have the advantage of being flexible, allowing them to follow the intravascular configuration of the pulmonary artery. Several designs have been proposed over the years, and the nitinol shape memory property provides wire kink resistance. The main reported loop snare technique is the proximal capture technique [5, 9–12, 15–17]. This simple technique allows for capturing the middle of the implant. We chose to perform this technique given the position of the proximal portion of the catheter in the left pulmonary artery. However, the use of a double-loop capture technique has been reported for extracting a device having migrated into a sub-segmental artery [13]. Due to the peripheral nature of the implant, the device was first captured using gentle traction at one end using a loop snare. A second femoral access was performed to secure the opposite end of the device, in order to align the long axis of the implant with the pulmonary outflow tract, avoiding wall lesion. This technique appears particularly suitable for treating peripheral migrations with partial endothelialization, provided there is space between the proximal end and the catheter. The forceps technique has historically been used but has become relatively obsolete since the advent of loop snares, and should not be used due to the risk of vascular wall injury.

**Table 1:** Reported cases of removal attempts

Cases	Pulmonary location	Endovascular/Surgical approach Type of technique	Succes/Failure of procedure	Complication
Gallon et al. [5] 2016	Lower right lobe Segmental artery	Endovascular approach Loop snare / Proximal capture technique	Success	No
Heudes et al. [10] 2015	Upper right lobe Segmental artery	Endovascular approach Loop snare / Proximal capture technique	Success	No
Maroteix et al. [11] 2015	Left pulmonary artery Segmental artery	Endovascular approach Loop snare / Proximal capture technique	Success	No
Akthar et al. [12] 2018	Lower right lobe Subsegmental artery	Endovascular approach Loop snare / Proximal capture technique	Success	No
Chung et al. [13] 2016	Lower left lobe Subsegmental artery	Endovascular approach Loop snare / Double capture technique	Success	No
Thomas et al. [4] 2016	Lower left lobe Segmental artery	Surgical retrieval Arteriotomy	Success	Hemothorax
D’Journo et al. [19] 2014	Lower left lobe Subsegmental artery	Surgical retrieval Trisegmentectomy	Success	No
Carraro et al. [14] 2021	Right lower lobe Subsegmental artery	Endovascular approach Aspiration	Success	No
O’Brien et al. [9] 2014	Left lower lobe Subsegmental artery	Endovascular approach Loop snare / Proximal capture technique	Failure Left in place	No
Mallak et al. [15] 2022	Left lower lobe Subsegmental artery	Endovascular approach Loop snare / Proximal capture technique	Failure Left in place	No
Gao et al. [16] 2018	Lower left lobe Subsegmental artery	Endovascular approach Loop snare / Proximal capture technique	Success	No
Wilcox et al. [17] 2018	Right lower lobe Segmental artery	Endovascular approach Loop snare / Proximal capture technique	Success	No
Carlos-Alves et al. [6] 2019	Lower left lobe Subsegmental artery	Thoracic surgery without pulmonary resection	Success	No
Wali et al. [18] 2014	Left lower lobe Subsegmental artery	Surgical approach Segmentectomy	Success	No

Once the device is captured, it can be retracted into the catheter. However, due to the potential risk of constriction and fragmentation of the catheter during its withdrawal into the introducer, we preferred to simply secure the device against the introducer and perform the retrieval under fluoroscopic guidance. Moreover, as the device is flexible (Fig. 1), the risk of injuring the vascular wall during retrieval appeared to be low. In our case, bi-planar fluoroscopic guidance facilitated the recapture of the contraceptive device. Endothelialisation of the device adhering to the vascular wall has been mentioned as a risk factor for technical failure of the endovascular approach [9].

The surgical approach consists of a segmentectomy, including the affected artery [4, 6, 18]. In one case, visualization of the device through the artery during thoracoscopy allowed for retrieval through arteriotomy, which was complicated by clot formation requiring pleural lavage the day after the procedure [4]. Reported surgeries were performed either after unsuccessful attempts at endovascular removal or because the device had migrated too distally to be retrieved endovascularly. However, due to the invasive nature of the surgical approach, we believe it should only be reserved in case endovascular removal failure, after a risk–benefit evaluation.

## Conclusion

The present case highlights the central role of multidisciplinary management in the removal of contraceptive implant by endovascular approach. The endovascular approach should be attempted first in a patient desiring contraceptive implant removal, as this minimally invasive approach is associated with a high rate of technical success without reported complications.

## Data Availability

Not applicable.

## References

[1] Berenson AB, Tan A, Hirth JM (2015). Complications and continuation rates associated with 2 types of long-acting contraception. American J Obstet Gynecol.

[2] Park JU, Bae HS, Lee SM (2017). Removal of a subdermal contraceptive implant (Implanon NXT) that migrated to the axilla by C-arm guidance: a case report and review of the literature. Med (Baltim).

[3] Ohannessian A, Levy A, Jaillant N (2019). A French survey of contraceptive implant migration to the pulmonary artery. Contraception.

[4] Thomas PA, Di Stefano D, Couteau C, D’Journo XB (2017). Contraceptive implant embolism into the pulmonary artery: thoracoscopic retrieval. Ann Thorac Surg.

[5] Gallon A, Fontarensky M, Chauffour C (2017). Looking for a lost subdermal contraceptive implant? Think about the pulmonary artery. Contraception.

[6] Carlos-Alves M, Gomes M, Abreu R, Pinheiro P (2019). Lung migration of contraceptive implanon NXT. BMJ Case Rep.

[7] Barlow-Evans R, Jaffer K, Balogun M (2017). Migration of a Nexplanon contraceptive implant to the pulmonary artery. BMJ Case Rep.

[8] Cerato A, Luyckx M, Ghaye B (2019). Migration of implanon contraceptive implant into the pulmonary artery. Diagn Interv Imaging.

[9] O’ Brien A, O’Reilly MK, Sugrue G (2015). Subdermal contraceptive implant embolism to a pulmonary artery. Ann Thorac Surg.

[10] Heudes P-M, Laigle Querat V, Darnis E (2015). Migration of a contraceptive subcutaneous device into the pulmonary artery. Report of a case. Case Rep Women’s Health.

[11] Maroteix P, Dupeyrat J, Roupie E (2015). Embolie pulmonaire par implant progestatif. Ann Fr Med Urgence.

[12] Akhtar MM, Bhan A, Lim ZY (2018). Percutaneous extraction of an embolized progesterone contraceptive implant from the pulmonary artery. Open Access J Contracept.

[13] Chung M, Loudill C, Wieler M (2017). Endovascular retrieval of nexplanon from the distal pulmonary artery. J Vasc Interv Radiol.

[14] Carraro do Nascimento V, De Villiers L, Chia GS, Rice H (2021). Aspiration technique for percutaneous endovascular retrieval of contraceptive device embolized to the pulmonary vasculature. Radiol Case Rep.

[15] Kafi Mallak F, Kopp Kallner H (2022). Migration of a subdermal contraceptive implant into a subsegmental pulmonary artery and etonogestrel serum concentration over time - a case report. Eur J Contracept Reprod Health Care.

[16] Gao GT, Binder W (2018). Embolization of a contraceptive implant into the pulmonary vasculature in an adolescent female. Am J Emerg Med..

[17] Wilcox KK, Turcer F, Soltes GD, Shin DS (2018). Endovascular retrieval of contraceptive implant embolized to pulmonary artery. Radiol Case Rep.

[18] Wali A, Bilkhu R, Rizzo V, Bille A (2021). Contraceptive implant migration to the lung. BJR Case Rep.

[19] D’Journo XB, Vidal V, Agostini A (2015). Intravascular pulmonary migration of a subdermal contraceptive implant. Ann Thorac Surg.

